# “I want to save my life”: Conceptions of cervical and breast cancer screening among urban immigrant women of South Asian and Chinese origin

**DOI:** 10.1186/s12889-016-3709-2

**Published:** 2016-10-13

**Authors:** Jennifer Hulme, Catherine Moravac, Farah Ahmad, Shelley Cleverly, Aisha Lofters, Ophira Ginsburg, Sheila Dunn

**Affiliations:** 1 Emergency Department, University Health Network, University of Toronto, Toronto, Canada; 2 Department of Family and Community Medicine, University of Toronto, Toronto, Canada; 3 Postgraduate Medical Education, University of Toronto, Toronto, Canada; 4 Faculty of Health, School of Health Policy and Management, York University, Toronto, Canada; 5 Dalla Lana School of Public Health, University of Toronto, Toronto, ON Canada; 6 Centre for Health Promotion, Department of Public Health Sciences, University of Toronto, Toronto, Canada; 7 Centre for Research on Inner City Health, Li Ka Shing Knowledge Institute, St. Michael’s Hospital, Toronto, Canada; 8 Department of Family and Community Medicine, St. Michael Hospital, Toronto, Canada; 9 Women’s College Research Institute, Toronto, Canada; 10 Department of Medicine, University of Toronto, Toronto, Canada; 11 Women’s College Hospital, Toronto, Canada

**Keywords:** Immigrant, South Asian, Chinese, Women, Access, Cancer, Screening, Behaviour, Qualitative, Policy, Health systems

## Abstract

**Background:**

Breast and cervical cancer screening rates remain low among immigrant women and those of low socioeconomic status. The Cancer Awareness: Ready for Education and Screening (CARES) project ran a peer-led multi-lingual educational program between 2012 and 2014 to reach under and never-screened women in Central Toronto, where breast and cervical cancer screening rates remain low.

The objective of this qualitative study was to better understand how Chinese and South Asian immigrants – the largest and most under-screened immigrant groups according to national and provincial statistics - conceive of breast and cervical cancer screening. We explored their experiences with screening to date. We explicitly inquired about their perceptions of the health care system, their screening experiences with family physicians and strategies that would support screening in their communities.

**Methods:**

We conducted 22 individual interviews and two focus groups in Bengali and Mandarin with participants who had attended CARES educational sessions. Transcripts were coded through an iterative constant comparative and interpretative approach.

**Results:**

Themes fell into five major, overlapping domains: risk perception and concepts of preventative health and screening; health system engagement and the embedded experience with screening; fear of cancer and procedural pain; self-efficacy, obligation, and willingness to be screened; newcomer barriers and competing priorities. These domains all overlap, and contribute to screening behaviours. Immigrant women experienced a number of barriers to screening related to ‘navigating newness’, including transportation, language barriers, arrangements for time off work and childcare. Fear of screening and fear of cancer took many forms; painful or traumatic encounters with screening were described. Female gender of the provider was paramount for both groups. Newly screened South Asian women were reassured by their first encounter with screening. Some Chinese women preferred the anonymous screening options available in China. Women generally endorsed a willingness to be screened, and even offered to organize women in their community hubs to access screening.

**Conclusions:**

The experience of South Asian and Chinese immigrant women suggests that under and never-screened newcomers may be effectively integrated into screening programs through existing primary care networks, cultural-group specific outreach, and expanding access to convenient community -based screening.

## Background

Despite well-established, organized cancer screening programs (Appendix 1) [1], new immigrants to Canada continue to be under screened for breast and cervical cancer [2–10]. The province of Ontario is home to the highest proportion of new Canadian immigrants and in Toronto, Ontario’s largest city, foreign-born Canadians make up 46.0 % of the population [11]. Only half (53.1 %) of urban immigrant women in Ontario were screened for cervical cancer between 2006 and 2008 [12] compared to 63.6 % of all urban female Ontario residents during the same time period [7]. Fifty seven percent of recent immigrants were found to be ‘non-users’ of mammography within five years of arrival to Canada compared to 26 % of Canadian-born women [3, 13].

Barriers to screening among immigrant groups are multifold [14–19]. Women who are less proficient in an official language are less likely to be screened, as are women originating from a region with low screening rates [5, 20, 21]. System and physician-level factors often compound individual barriers. Women seeing a physician from their region of origin are less likely to be screened, [12, 22] and South Asian-trained physicians are even less likely to screen South Asian women for cervical cancer [4, 21]. Access to a female provider and a family physician remain important determinants of screening [4, 7, 12, 14, 23–28].

This evidence formed the basis for the Cancer Awareness: Ready for Education and Screening (CARES) project, a collaboration of over sixty community-based organizations and two academic hospitals in Toronto. The project aimed to reach facilitate access to breast and cervical cancer screening through various networks for new immigrants, refugees, and the under housed in Toronto. At the core of the project were 42 Peer Leaders, who were recruited through partner agencies to provide health education workshops in their native languages.

Women of South Asian and Chinese descent made up a large proportion of CARES program participants, and are the most important immigrant groups in Canada [11]. They are also among the most likely groups to be under or never screened for breast and cervical cancer [5, 29, 30]. The current literature mostly aims to identify predictors of screening behavior and cultural-specific barriers to screening [31–34]. Less is know about the shared experiences of new immigrants in North America in seeking and obtaining screening, and their interaction with the health system.

In this qualitative study, we obtained a purposeful sample of Mandarin and Bengali speakers to better understand of how two groups conceive of breast and cervical cancer screening. Our research questions aimed to illuminate participants’ perspectives on:

- ongoing knowledge gaps and barriers related to breast and cervical cancer screening
- the role of family physicians and primary care providers in screening; and
- solutions and preferences to facilitate future access to screening for women in their communities

This work forms part of a wider initiative in Ontario to identify the key components of an effective regional program for breast and cervical cancer screening [35].

## Methods

Between January and April 2014, we held focus group and individual interviews with a purposeful sample of Mandarin and Bengali speakers to explore their fears, expectations, and experiences to date, as well as their perspective on why women in their communities may not access screening. We explicitly asked about their experiences with Pap tests and mammography in Canada, and about their experience with family physicians, which are the major providers of screening in Ontario. Their perspectives and preferences were also gathered to identify possible solutions to facilitate access to screening among their peers and in their community. Preliminary semi-structured interview guides were developed and refined by the study investigators to reflect these research questions, and further revised based on Peer Leaders’ feedback. They were then translated into Mandarin and Bengali and back translated into English. This study used a grounded theory approach based on Glaser and Strauss [36]; the analytical approach was influenced by the principles and strategies espoused by Kathy Charmaz [37]. A combination of focus group and individual interviews allowed a broad range of themes, which were then further explored in focus group discussions. It also allowed inclusion of a more diverse group of participants whose availability and comfort with the two methods might differ.

From May 2012 to October 2014, CARES held 148 educational sessions conducted in 20 languages for 2023 women and facilitated 161 Pap tests and 88 mammograms for attendees. We recruited interview subjects from the pool of 739 CARES participants who agreed to receive a follow-up phone call. Mandarin and Bengali speakers who were 4–12 months post-education were telephoned and invited to participate.

Two bilingual Peer Leaders were selected for their interviewing skills to conduct telephone interviews and trained by two co-authors (JH, CM) in non-judgmental, open-ended interview techniques. JH conducted interviews in English as a second language with other CARES participants using the same methods and semi-structured questionnaire. We included two English interviews with women from Bangladesh in this data set. At least three attempts at telephone contact were made before removal from the participant pool. Phone interviewees were offered token gift certificates for their time. Verbal informed consent was obtained from each participant over the phone [38]. Individual interviews were 20–65 min long.

All interviews were transcribed and pseudonyms were used during transcription to ensure anonymity. One-on-one Bengali and Mandarin interviews transcribed and translated by the same bilingual Peer Leaders that conducted the interview. English language interviews were professionally transcribed.

To add to the analysis, focus groups were also conducted with CARES participants. CARES Peer Leaders openly invited Mandarin and Bengali speaking CARES participants to participate in focus groups at partnering community centers. A focus group interview guide was developed (JH, CM) to provide further insight on the lived experiences of these women. Participants were offered two-way tickets for public transport and light refreshments. Two bilingual, professional facilitators with no involvement with the CARES project were recruited and trained to moderate the Mandarin and Bengali-language focus groups. Written information consent was obtained from all focus group participants. Focus groups ranged from 45–75 min, which were audio recorded and field notes were taken. Focus groups were transcribed and translated by the bilingual focus group facilitators due to budget limitations.

All procedures were approved by the Women’s College Hospital Research Ethics Board and St. Michael’s Hospital Research Ethics Board.

### Data analysis

Multiple strategies were used to ensure rigor and trustworthiness of findings. After the initial reading of interview and focus group transcripts, notes were made in the margins. Deductive strategies were used to identify parent codes followed by child codes. The coding structure was initially identified through multiple readings of interview notes and transcripts by four separate reviewers and discussed to establish congruency (SD, FA, JH, CM). Two reviewers (JH, CM) subsequently organized and coded the transcripts through an iterative constant comparative and interpretative approach. Attention was paid to outlier codes as well as those that were congruent. Through an inductive process themes were identified and tested [39]. A third reviewer (SD) identified and triangulated the thematic analysis with the two main reviewers. Throughout the analysis, we noted findings that appeared to be specific to culture and country of origin. Data was organized using TamsAnalyzer® software [40].

## Results

We held two focus group discussions, one in Mandarin and the other in Bengali, and individual interviews with 23 women that yielded rich data and saturation of predominant themes. Available demographic information is outlined in Table 1; note that some demographic information was not available due to non-response. Most women were up to date on their screening either on their own, or through CARES program facilitation; this is a reflection of recruitment from the pool of CARES program participants, where fewer than half were under or never screened for cervical (44 %) and breast cancer (43 %). CARES program-specific outcomes are reported separately [41].

**Table 1:** Participant demographics

One on one interviews					
Country of origin	Number of participants (n)	Ages	Language of interview (n)	Time in Canada (n)	Screening status (n)
China	11	33–69 Median 55	Mandarin (11)	<5 years (3) ≥5 years (5) N/A (3)	Pap UTD (3) Never had Pap (2) Unsure (1) N/A (3)
					Over 50 Pap & Mammogram UTD (5) Pap UTD, Mammogram not UTD (1) Unsure (1)
Bangladesh	12	30–61 Median 39	Bengali (10) English (2)	<5 years (4) ≥5 years (5) N/A (3)	Pap UTD (6) Never had Pap (2)
					Over 50 Pap & Mammogram UTD (3) Pap UTD, Mammogram not UTD (1)
Focus groups					
Country of origin	Number of participants (n)	Ages	Language of focus group	Time in Canada (n)	Screening status (n)
China	6	28–65 Median 38	Mandarin (6)	N/A	N/A
Bangladesh	8	34–65 Median 42	Bengali (8)	<5 years (3) ≥5 years (2) N/A (3)	UTD Pap (1) Never had Pap (2) Unsure (1) N/A (2)
					Over 50 Pap UTD, Mammogram not UTD (1) N/A (1)

*Abbreviations*: *N/A* Data not available due to non-response; *UTD* Up to date, *Pap* Papanicolauouo smear (cervical cancer screening)

Five major themes emerged from the focus group discussions and interviews. The common themes encountered were grouped under five non-hierarchical domains:

- risk perception and concepts of preventative health and screening
- health system engagement and the embedded experience with screening
- fear of cancer and procedural pain
- self-efficacy, obligation and willingness to be screened
- newcomer barriers and competing priorities

Other themes emerged that appeared specific to Chinese and South Asian groups are interwoven and highlighted through these groupings. These domains overlap and interact on multiple levels, and contribute to participants’ overall attitudes, beliefs, motivation and willingness to be screened.

### Risk perception and concepts of preventative health and screening

**Screening was a challenging concept**. A number of women specifically cited the absence of symptoms as a reason not to get screened. “My health is pretty good, there is nothing to be concerned about”. Cyclical breast pain and ‘lumpy breasts” were also described as worrisome and a reason to seek screening, and participants were unclear as to the role of mammography in addressing these symptoms.

Several informants described their anxiety or confusion about their medical investigations for abnormal uterine bleeding or pelvic pain, and remained uncertain about the purpose of Pap tests, citing their purpose to identify medical conditions such as uterine polyps and fibroids. There were also a few instances in which Pap tests were postponed until the presentation of symptoms.

“I feel that Pap test cannot find anything. Every time when I went to have Pap test, they told me everything was fine. Afterwards, when I had the ultrasound - you know because I bleed too much - they told me they found three…. polyps.

*55-year-old Mandarin speaker, in Canada for greater than 5 years, Pap and Mammogram up to date*

“Different reason, sometimes we may think our health is pretty good. We do not feel any big change for our health condition, and at the same time, we are too busy” – *participant #3*

“For young people like us, we may feel we are fine, not any serious problem with our health, even if there is minor problem, we can recover by ourselves, so , ok,, that is it, that is it, do not worry. Later I can go to the doctor and see what is the problem, I will wait till I have to have the test.” – *participant #4*

*Mandarin focus group*

**The strengths and limitations of screening** were generally not well understood, and this translated to potentially false reassurance of perfect health, or being cancer-free. Absolute terms were sometimes used to describe the benefits of screening: “My doubt was eliminated after my mammogram.” “I do not have disease, I have just finished my check up.” “I want to save my life!”

**Risk perception** was shaped by an individual’s personal experience with health, ill health and screening to date, as well as contact with family members and acquaintances that had been diagnosed with cancer of any kind.

“…In Bangladesh I did not see or hear our guardians or elders talk about Pap test. I know so far this is for ovarian cancer or cervical cancer! …My family does not have this history so why I should do it unless I have any problem… … because I am not feeling any problem I am not interested to do the Pap test.”

*46-year-old Bengali speaker, in Canada for greater than 5 years, never had Pap*

### Health systems engagement and the embedded experience with screening

Easy access to screening was generally credited to **no-cost access and relative ease of accessing health practitioners** in urban Canada.

“Yes, after [the CARES session] I went to… my family physician, and did all the related tests that you mentioned at the presentation. We all want to be safe and because treatment here is free, and I came to know from your program that there is no pain and no problem in doing it, so I became interested.”

*30-year-old Bengali speaker, in Canada more than 5 years, Pap up to date*

“If…the doctor wants me to do that, I will do it, it is covered by [provincial insurance], right? After I deliver my baby, when I go to my doctor for regular visit, I will check with him when he will arrange the PAP test for me. .(Probe)…. Well, back in China, no, because in China the government will not pay for us, it seems no one will go to do this test unless if they do not feel well, they will not go for regular screening. Wait, yes, unless we do it though our annual check up which is organized by our company.”

*33 year old Mandarin speaker, in Canada less than 5 years, unclear if Pap up to date but recently obtained in China*

We specifically asked about the **role of primary care** providers in screening. Language barriers, and feeling **rushed or not heard** by their providers were vividly described by a few respondents.

“My family doctor, well, I’m not willing to talk to him. He is not professional, very bad. Every time when I go to see him, he just finishes in several minutes. He is not at all patient. Also, he is a male doctor; I do not want to have a check up with him at all. My previous mammogram was done in Etobicoke, the doctor seemed…. not very professional, his attitude was not nice, in one word, I was afraid.”

*52-year-old Mandarin speaker, unknown time in Canada, Pap up to date, requesting help from CARES to obtain mammogram*

“….Here doctors don’t give time, when I go then I have to wait for 2 h but they spend only 10 min with me. They don’t take care about what patient needs to know about pap.”

*58-year-old Bengali speaker, in Canada 5 or more years, screening up to date*

Women strongly voiced their preference to **be informed of the result** of the mammogram or Pap test, even if it is normal. Notably, this practice has since changed in Ontario, and patients receive a letter in the mail with their result.

“People always say, no news is good news, but there is always a concern in my heart, why not tell me the result”

*64-year-old Mandarin-speaker, in Canada more than 5 years, Mammogram up to date, not eligible for Pap*

“My opinion is that the family doctor should inform her patients about their any test result… whether it is positive or negative, it is my right to have a copy.”

*61-year-old Bengali speaker, in Canada for less than 5 years, screening up to date*

**Family physicians were otherwise viewed very positively**. Bengali-speakers especially valued the convenience, continuity and support offered by their providers, and felt that their reminders were effective in prompting them to obtain both cervical and breast cancer screening.

“It’s good. Here in this Canada I see that family doctors are … they are someone to coordinate all of my health problems. So it’s good, family doctor… they try to handle this.”

*33-year-old Bengali speaker, unknown time in Canada, Pap up to date through CARES*

“I feel comfortable with my family doctor. Yes I can talk with the doctor freely and don’t face any problem

*58-year-old Bengali speaker, in Canada greater than 5 years, screening up to date*

“.. is more convenient, because…they can have the general check up together with the Pap test there, they will like to do it together…. you do not need to make another arrangement, and you do not need to have a separate travel. You know, you have to ask for a leave from work for that, right?

*35-year-old Mandarin speaker, in Canada unknown amount of time, Pap up to date*

There were some differences in the role of the family physician in promoting cervical cancer and breast cancer screening. Generally, respondents followed their physician’s advice to obtain mammography. Obtaining Pap smears depended more on the gender of the provider, ease of access, and the other factors described in this study. The importance of a physician **recommending and facilitating screening** was reiterated with examples of **opportunistic screening** initiated by their family physician. Once women were engaged, they were often retained in screening if they had a positive experience.

“Because I had an itchy vagina, then the doctor asked me whether I have ever done a Pap test, I told him no… the doctor asked me, “can I do your pap test?” Then my family doctor told me to go for Pap test with this female doctor. Since then I go by myself to do my pap test.”

*37-year-old Bengali speaker, in Canada more than 5 years, Pap up to date*

**Female gender of the health care practitioners** was paramount in facilitating access to cervical cancer screening for almost every informant. Some expressed frustration with the lack of choice in Canada in accessing female practitioners – “the female doctor is really very…very few… right?”. Others reported that they had been screened after their doctors referred them to a female practitioner for their Pap test.

“…we Bengali want to go to a female doctor to do a Pap test, but some doctors are male doctor and women do not want to do their Pap with them… For [women] who have a male family doctor, if a nurse or doctor from [other institutions] can come and do this test, it would be better.”

*30-year-old Bengali speaker, in Canada more than 5 years, Pap up to date*

“Because my family doctor is a male doctor, I could not find a female doctor, I know… this person who refused to be checked by male doctor. So she had never had one test. I told her if she had attended your session, your project could have find female doctor for her.”

*56-year-old Mandarin speaker, in Canada greater than 5 years, screening up to date*

Bangladeshi informants described their preference for female providers in terms of **modesty**. Chinese respondents described **embarrassment or shame** with the prospect of having a Pap, which is worsened still if done by a male provider. In contrast to Bengali informants, who often voiced their preference for a relationship with their primary care provider, Chinese informants often coupled preference for a female provider with asking Peer Leaders for a referral to an anonymous, third party option for care.

“…today I felt so embarrassed, I did not feel comfortable, because I was checked by a male doctor today. Yes, if there is workshop in the community, this will… encourage me to have the check, why, well, you know, because all this check up is embarrassing, right? (Hesitates) … because we are all married, it should be ok for them to have this check, but in another way, it is also embarrassing for you to tell others that you are having this kind of check up. You can not tell them, ‘oh, I have gone for a check up today, it was done by a male doctor’.”

*44-year-old Mandarin speaker, in Canada unknown time, Pap up to date, had mammogram*

Participants’ experience with the **health systems in their home countries** strongly influenced their attitude towards screening in Canada, as well as their level of confidence in their new health care system. Participants from Bangladesh emphasized that screening was often a new concept for them that they encountered for the first time in Canada, unless they were aware of the test but were unwilling to pay out of pocket in Bangladesh. Informants from Bangladesh specifically contrasted their experience with that of their health system in their country of origin, often highlighting the **relative ease of access to care now,** and were often engaged in screening for the first time through their primary provider.

In contrast, Chinese women of all ages had been exposed to screening in China. “I often have this checked up back in China, not only here, preventive check up.” Chinese informants were often predominantly frustrated with the gatekeeper system restricting access to specialists, and slow access to diagnostics – “Back in china, if today your disease is diagnosed, tomorrow the hospital can arrange a surgery for you.” This did not necessarily deter them from screening, but they expressed a preference for hospital-based specialist delivered care, or cited positive experiences with company-sponsored employee annual health checks in China, and a preference for group screening activities.

“I hope you can help to arrange that for me, I trust more for the hospital for checking up…. over some other small place, I do not know why, I just do not have confidence for them.”

*64-year-old Mandarin speaker, in Canada greater than 5 years, not eligible for Pap, mammogram up to date*

### Fear of cancer and procedural pain

Fear of cancer and fear of a positive test result were common among both South Asians and Chinese informants. Some had become anxious about their risk through external influences like the media’s intensive coverage of breast cancer, or through the unintended consequence of the CARES program itself. A few participants avoided screening altogether to avoid finding out the unknown.

“Very concerned…. because, because some symptom might a little bit similar to the cancer, so I feel I am afraid. Sometimes, this kind of concern may be not necessary, but… I do not feel like going for a check, I do not like check. I am kind of the person that do not like to go for check even if I am sick, the feeling to be final judged by the doctor, I do not like that, so I do not like go to see the doctor.”

*52 year old Mandarin-speaker, unknown time in Canada*, *Pap up to date, requested help from CARES for mammogram*

“Before I attended the info session, I seldom paid attention to the check up or whatever, neither did I pay attention to the talks in the TV, radio. Now I try to pay more attention to that, but the more I learn, the more I am scared…It is better for me not to know so much…I would be less nervous if I did not know so much. I am so stressed.”

*56-year-old Mandarin speaker, in Canada greater than 5 years, screening up to date*

Fear was a central theme, and was related to various aspects of cancer screening. Fear of the procedure itself was common, and often linked to pain in the context of mammography. Fear and dread was heightened if there was no explanation of the procedure before it was done. Several informants described specific physical and emotional trauma incurred by an aggressive practitioner, and a rushed or painful Pap test by an unskillful practitioner, and this negatively influenced willingness to be screened again. While this finding was more commonly described for mammograms, it highlights the effect of certain unhelpful approaches in negative experiences and in undermining willingness to be screened for both procedures.

“I just had it checked two years ago; because I feel it is too painful, I do not want to check it anymore. Right, when the machine compressed, it was very painful. … I feel they should be gentle… I mean I did it in [urban location], the female doctor dragged my breast here, there, very hard, I feel it is unbearable, so I do not want to check it anymore.”

*60-year-old Mandarin-speaker, in Canada more than 5 years, not eligible for Pap, Mammogram 2 years ago but unwilling to do again*

A number of sources of anxiety were specific to Chinese informants. One of the most striking examples was the concern about the **sanitation of medical instruments**. “I am afraid that [the speculum] is not properly sanitized. It is no good. A healthy person might catch disease through this.” “Before they start the Pap test, they can explain to the patient if the instrument is disposable or if it is not, have it been well sanitized”.

A number of Chinese informants described ‘painful’ and ‘scary’ **invasive diagnostic procedures** as a result of breast lumps or breast complaints. This diagnostic experience became an ongoing source of unease about their overall health. Rarely did these experiences take place in the context of routine screening, but rather in response to common breast complaints that had led to invasive testing both in China and in Canada.

“….after your session, I have went to re-check my breasts, after my previous surgery, I am kind of rejecting the doctors in Canada, en, because, first, he cut open my breast, and told me there is something inside, then the biopsy, and could not tell what was that, He told me it might be cancer, it might be not, you made your own decision whether you want to remove it.”

*44-year-old Mandarin speaker, in Canada unknown time, Pap up to date, had mammogram*

Many participants from Bangladesh were never screened for either breast or cervical cancer until arrival to Canada. They described their dread leading up to screening, which dissipated after participating in a positive screening experience, or a process of ‘**normalization**’.

“[The first time] I was feeling scared about what would happen, then that neighbour doctor said, you will have nothing, everything would be normal…. Then I did it. At first I got a little fear but after that it’s become normal. Now, when I am asked for an appointment, I try to make that very day when they ask. Sister… honestly when I went for the first time….I went with so many layers of my clothes due to winter and I have to put them down from top and I felt uneasy to put down clothes…laughing…at first….you know we are Bengali…then I felt uneasy though female was there…after then I got used to it and I used to dress for the test friendly. The first time I did not have a vehicle, so it was not easy, but it became easy now when my kids drop me. Also my husband does… There is no problem that I feel when I need to do something important.”

*52-year-old Bengali speaker, in Canada more than 5 years, screening up to date*

“Interviewer: Easy, okay. And what makes it easier? What do you like about the system?

Respondent: And I didn’t feel anything. I heard that it will be painful… but it was not painful, it was okay. I am happy with that.”

*33-year-old Bangladeshi, unknown time in Canada, Pap up to date*.

These positive experiences helped them to overcome their fear and supported their willingness to participate in future screening. A gentle operator and clear explanation of the procedure beforehand was also described in reducing anxiety before and after the procedure.

“When I did my pap then there was a female doctor, not from our community …then she told me before the test. Here doctors usually say about the procedure before the test so it helps in reducing fears. Because she told me before so I did not have fear.”

*37-year-old Bengali-speaker, in Canada more than 5 years, Pap up to dat*e

### Self-efficacy, obligation to others, and willingness to be screened

Most respondents were willing to be screened, irrespective of their age or screening status, and described screening as a socially desirable act viewed positively, with internal and external rewards. The exceptions were those that had a traumatic screening experience, internalized fear leading to complete avoidance, or whose risk perception was exceptionally low.

This willingness to be screened was expressed in terms of **self-efficacy** - “doing something for my health” – “I will consider my own health condition”. While these reflect some of the CARES program messages around gender and self-care, the strength and pervasiveness of these statements suggest internalized concepts of self-determination. We specifically inquired about the influence of family members, such as husbands, in determining whether women can go or are supported to go for screening. A few Bengali informants knew of other women whose husbands would deter them from seeking screening, but the participants themselves described their families as supportive, helpful with transportation or translation if needed, and supportive of the decision to obtain screening as their own. “I make the decision by myself for everything”. Bengali-speaking participants actively offered to organize other women in their apartment building and their community to obtain further education sessions, or organize group-screening events. This enthusiasm was also reflected in the terms used in relation to screening among younger participants, that screening provided “reassurance”, that they ”want to be healthy”, and screening will “save my life”:

“You need to love yourself, then you can love others, if you do not love your self, can you expect others love you.”

*35-year-old Mandarin-speaker, unknown time in Canada, Pap up to date*

Motivation was also described in terms of **obligation and duty to their family stay healthy.** “For the sake of your happy life, your family’s happy life… for the sake of good health” (*Mandarin focus group*). Younger Chinese and Bangladeshi informants also endorsed screening as part of their duty as mothers.

“Right, this is also good for Canada, if all these group women were left alone, they are all mothers, you know, the emotion of mom can affect their kids, the wellbeing of the next generation in Canada can be affected, right?”

*44 year old Mandarin speaker, unknown time in Canada, Pap up to date*

“Now that I have a baby, no one’s going to look after her if her mom dies, it’s finished….” – *Participant #3*

“Oh please don’t mention it!” - *Moderator*

“So, I have to live for my daughter. I have to work for her” - *Participant #3*

*Bengali focus group*

Older Chinese women voiced an often-grudging obligation to follow their family or doctor’s recommendation.

“When it was done? Very nervous, pretty afraid. Also very uncomfortable, I do not like this kind of check up, but the doctor wanted me to do it, so I did it considering of my age. Good thing is that the result is normal. … (Sighs), for health, if it is needed, I have to do it.”

*52-year-old Mandarin speaker, unknown time in Canada, Pap up to date, Mammogram out of date, requested help from CARES*

“For me, if I were alone, I am lazy, I do not feel like going for the check up… My children, my family members, on the other hand, feel that I need to have this check up…. they believe that if I will not go for the check up, that might lead to even bigger problem.”

*55-year-old Mandarin speaker, in Canada for more than 5 years, Pap and mammogram up to date*

### Newcomer barriers and competing priorities

Multiple, parallel barriers often coexisted in navigating a new heath system, transportation, childcare, time away from work, and language. **Language barriers** were a central theme in ‘navigating newness’, and often necessitated accompaniment to visits by younger, bilingual family members who could translate. “I cannot speak English, if I go myself… I have to have my daughter with me, right?”

“The doctor gave us a date, even my husband did not know about the place where to go. We went over there and waited for one hour or one and half, after they saw us. Travelling there is also a problem. We are new here and we don’t know anything and where we should leave our kids to take care…. The doctor- his language and my language, could not communicate …I could not describe my problem accurately…. Now I am communicating with you in Bengali and I am sharing everything that I have in my mind - but when I go to doctor then I have to speak in English, and I cannot speak English clearly. Whatever I told her, she understood differently; and what she said, I understood something else; in that context, we cannot express ourselves freely.””

*35-year-old Bengali-speaker, in Canada less than 5 years, Pap up to date through CARES*

“I don’t have small kids and my son is grown up so I did not feel that hard. If it was far from me, or.... If the doctor spoke another language then I would have a problem. Sometimes Chinese doctors don't understand me, nor can I understand them. Here my doctor was from my community and even the assistant was from my community so I did not feel any problem..”

*40-year-old Bengali speaker, in Canada less than 5 years, Pap up to date through CARES*

**Competing priorities with work and childcare** were of greatest concern for young Chinese mothers, whereas **transportation, language, and cultural barriers** were the challenges voiced by older unilingual Chinese women. Younger South Asian mothers also described limited mobility with their young children, especially during the Canadian winter as well as loneliness and **social isolation**. “I can not just get rid of the influence from the bad emotion, our social networking is so narrow, besides kids, there is only family - we are confined.” “Because women from our community have shyness, less familiar with travelling alone, and don’t want to go out.”

“I don’t know a lot of people here. I am newcomer. After that I got pregnant, I had a baby. I am almost confined in the home all the time.”

*33-year-old Bangladeshi respondent, unknown time in Canada, Pap up to date.*

In order to address these newcomer barriers and limited mobility of young mothers, informants of diverse age and culture suggested that primary care and screening outreach should be based in **community hubs** (i.e. apartment complexes and community centers) where newcomers could have screening services provided in their own language. They cited a tradition of community and group gatherings, and limited access to outside media, especially among older Chinese participants in support of this. Moving screening closer to the community would allow for older community members to come together socially, and for the younger women to assist each other with childcare. While the CARES peer leader model may have influenced this finding, some Mandarin speakers felt screening access is facilitated by having a Mandarin-speaking peer leader to guide the process, help with paper work and explain the screening procedures.

“Last time, in Universal [University] Settlement, they have a mobile health bus, parking in the park, helped to check us, I feel it is very convenient.”

*60-year-old mandarin speaker, in Canada greater than 5 years, not eligible for Pap, Mammogram up to date*

“… it is better to make things convenient for women with kids. It will be ideal if we can just have it in the community, in nearby. If there is someone help with the kids, if will be easier. It is easier to be a daddy… To bring the kids to the community, there is someone helping to take care of the kids there, so we can socialize at the same time…”

*35-year-old Mandarin speaker, in Canada unknown time, Pap up to date*

“If you go to Bengali community, school, mosque, door to door. I think these two sectors are very important. You will get a good chance to talk with others or come closer to them. Yes, Mosques---my kids go to learn their Islamic education on Saturday and Sunday. When class ends then you will get some mothers there, also at schools after the class you will also get many mothers.”

*35-year-old Bengali-speaker, up to date on screening after CARES project, in Canada less than 5 years*

**Generational differences** were also highlighted in the context of multi-pronged approaches to reach other Chinese women in their communities. For example, communication strategies differed with age: younger Chinese women said they navigate the Internet and ask their peers for health information, whereas older Chinese women reported use of the radio. Language issues were magnified for older Chinese women.

“I feel that kind of workshop is easier to understand. Also for people at this age, their English is not good. Because people who have high risk of cancer usually are not young people. Most of them are over 50. For people over 50, they do not speak good English, they do not know how to use the internet…Workshop and telephone communication is better way.”

*56-year-old Mandarin speaker, in Canada more than 5 years, screening up to date*

## Discussion

Immigrant women in large urban centers have many insights to offer in relation to mammography and cervical cancer screening programs for women in their communities. Gaps in biomedical understanding of the strengths and limitations of screening, confusion of screening with testing for common breast complaints, and the unintended consequence of cancer-related worry, anxiety, and increased perception of cancer risk are not in any way unique to new immigrant groups [42–46] However this study highlights specific areas of vulnerability among two major immigrant groups in terms of language, cultural and logistical barriers, as well as opportunities for health systems improvements and effective outreach and communication strategies.

A number of the ethno-specific findings on care-seeking beliefs and practices add to the existing literature on Chinese and South Asian groups in North America [47, 48]. Disparities between the Canadian and Chinese health care infrastructures were striking. In some cases, Chinese women preferred depersonalized, ‘third party’ Pap tests, carried out in large hospitals or mobile health units organized by their employer. Chang et al. postulate that anonymity with Pap testing may also lessen the embarrassment and sexuality attached to getting a Pap test and thus encourage its usage among Chinese women [33]. Mammograms were not overtly shameful, but a source of pain and discomfort which women were mostly willing to endure if their physicians or family recommended it, a finding echoed in the literature [49]. In contrast, Bangladeshi informants had rarely encountered screening their home country and expressed overt gratitude for their new health care system; this finding may be in part a reflection of differences in socio-economic status that we didn’t measure. Pap tests were enshrouded in similar apprehension and embarrassment, a positive experience with a patient provider often dispelled their concerns. They also highly valued rapport with their provider, a more typical predictor of ‘patient adherence’ [50]. Both groups shared similar fears about cancer and pain, and were motivated by a balance of obligation, self-efficacy, and a desire to be healthy.

Important opportunities for improving screening practices exist within the primary health care system. This study highlights the essential role of family physicians in supporting women in cancer screening. Opportunistic screening recommended by their primary care provider had been effective for a number of informants, demonstrating its importance in newcomer populations [21]. Although many women described fear and anxiety around cancer screening, for some, screening was normalized after the first, positive screening encounter. This highlights the importance of engaging women into the screening system for the first time with a positive screening experience. Careful explanation of the procedure and side effects, a gentle approach to the exam, and appropriate use of translators were all highlighted as key elements of strong practice. Physician gender preference patterns will remain a critical determinant of screening [4, 12]. The feminization of medicine, group family practices, and the rise of nurse-led screening programs may further facilitate access for women. Given physician and systems-level barriers to screening, alternative entry points, including mobile, walk-in and women’s health clinics would further facilitate timely access to female providers. They would also serve to honor Chinese participants preference for ‘anonymous’ testing.

A number of policy and planning priorities emerged from this study. A passive system historically relied on individuals to seek out both a primary care provider and screening. The Ontario Cervical Screening Program now sends reminders to women who have never had or are due for screening [51]. Letters are available only in English and French, however, which may be inaccessible to many newcomers. Our research and other studies suggests this may be less effective in increasing screening uptake than the general population [52]. Our research suggests that a more proactive, community-based model for seeking out and supporting newcomer women is still needed. The gendered barriers of balancing paid work, childcare and dependence on family members for transportation and language support still limit access for many women. Respondents across age and cultural groups explicitly expressed preference for the convenience and familiarity of nearby community-based screening and education sessions. Programs are often geared toward whole immigrant groups, and there are risks in attributing low uptake of service to cultural difference. The same strategic interventions may therefore be broadly effective across diverse groups of new immigrants. Community hubs, mobile health units, and employee or community-based ‘screening days’ were also suggested to address the time and financial burden of time off work and child-care costs. The strengths and limitations of the peer leader model employed in the CARES program will be described elsewhere, but certainly incorporating breast, cervical and colorectal cancer information sessions into natural meeting points and existing programs for newcomers or immigrants is an inexpensive, viable strategy that was very well received.

### Limitations

There are a number of important limitations of this study. The predominant focus on two major immigrant groups provides results that may be most relevant to these groups. Resources are limited, however, and by exploring how their experiences are similar, we can help inform health system reform and program evaluations, and develop a general public health approach to reaching these groups. The majority our respondents were in fact up-to-date for their screening, a reflection of the selection bias of CARES program participants who were more likely to have a positive view of screening given their participation. Notwithstanding, all informants offered a window to their experience with screening and the perceptions of women in their community about screening. The influence of the CARES project itself could not always be controlled for. CARES peer leaders interviewed women, and the positive aspects of screening may be over stated due to social desirability bias [53]. However, the richness of the data was likely due at least in part to the common language and trusting relationships our informants forged with the peer leaders. We addressed this by openly highlighting the likely areas of influence of the CARES program, and through our efforts to report the most transferable findings from our interviews.

## Conclusion

This study of women of South Asian and Chinese origin provides valuable insight into the barriers and opportunities to improve cancer-screening coverage for all newcomers and existing under and never screened groups. Priority should be given to cultural-group specific outreach, opportunistic screening through existing primary care networks, and expanding points of entry to convenient community -based screening modalities.

## Appendix 1: Breast, cervical and colon cancer screening program summary for Ontario, Canada

### Breast cancer screening

Women 50 years old and older are eligible for breast cancer screening with the Ontario Breast Screening Program (OBSP) and may refer themselves to the OBSP for a mammography. Women between the ages of 30 to 49 who are at high risk for breast cancer are also eligible but need a referral from their doctor or nurse practitioner to be screened through the OBSP. Women are recommended to have a mammogram every 2–3 years. Yearly mammograms are recommended for women at high risk for breast cancer. Mammograms are covered under provincial insurance.

https://www.cancercare.on.ca/pcs/screening/breastscreening/breasthealth/

### Cervical cancer screening

Women 21 year old and older, who have been sexually active, are eligible for cervical cancer screening every three years, unless their health care provider recommends more frequent screening. Women must make an appointment with their clinician for pap testing, which is currently the only cervical cancer screening test covered by provincial health insurance.

https://www.cancercare.on.ca/pcs/screening/cervscreening/screening_guidelines/

## Abbreviations

N/A: Data not available due to non-response
Pap: Papanicolauouo smear (cervical cancer screening)
UTD: Up to date.
